# Nectary tracks as pollinator manipulators: The pollination ecology of *Swertia bimaculata* (Gentianaceae)

**DOI:** 10.1002/ece3.3838

**Published:** 2018-02-19

**Authors:** Shuai Wang, Wen‐Long Fu, Wei Du, Qi Zhang, Ya Li, Yu‐Shu Lyu, Xiao‐Fan Wang

**Affiliations:** ^1^ College of Life Sciences Wuhan University Wuhan China; ^2^ Laboratory of Aquatic Plant Biology Wuhan Botanical Garden Chinese Academy of Sciences Wuhan China; ^3^ University of Chinese Academy of Sciences Beijing China

**Keywords:** circling behavior, nectary tracks, pollinator manipulation, postarrival filtration

## Abstract

Floral nectaries are closely associated with biotic pollination, and the nectar produced by corolla nectaries is generally enclosed in floral structures. Although some *Swertia* spp. (Gentianaceae), including *S. bimaculata*, evolved a peculiar form of corolla nectaries (known as “gland patches”) arranged in a conspicuous ring on the rotate corolla and that completely expose their nectar, little is known about the pollination of these plants. Two hypotheses were made concerning the possible effects of gland patches: visual attraction and visitor manipulation. The floral traits, mating system, and insect pollination of *S. bimaculata* were examined, and the pollination effects of gland patches were evaluated. A comparative study was made using *Swertia kouitchensis*, a species with fimbriate nectaries. *Swertia bimaculata* flowers were protandrous, with obvious stamen movement leading to herkogamy in the female phase and to a significant reduction in nectary–anther distance. The species is strongly entomophilous and facultatively xenogamous. The daily reward provided per flower decreased significantly after the male phase. The most effective pollinators were large dipterans, and the visiting proportion of Diptera was significantly higher in *S. bimaculata* than in *S. kouitchensis*. Most visitors performed “circling behavior” in *S. bimaculata* flowers. Removing or blocking the nectaries caused no reduction in visiting frequency but a significant reduction in visit duration, interrupting the circling behavior. The circling behavior was encouraged by nectar abundance and promoted pollen dispersal. Visitor species with small body size had little chance to contact the anthers or stigma, revealing a filtration effect exerted by the floral design. These results rejected the “visual attraction” hypothesis and supported the “visitor manipulation” hypothesis. The nectary whorl within a flower acted like a ring‐shaped track that urged nectar foragers to circle on the corolla, making pollination in *S. bimaculata* flowers more orderly and selective than that in classically generalist flowers.

## 1 INTRODUCTION

Floral nectaries are specialized structures in angiosperms that are closely associated with biotic pollination (Nicolson, Nepi, & Pacini, 2007). Nectar is easy for plants to produce and easy for animals to intake and digest, and it is often the primary offering of a flower, enhancing the reproductive success of plants (Willmer, 2011). Plants invest substantial amounts of sugar and water to supply nectar as a readily available energy source for animals (De la Barrera & Nobel, 2004), which makes nectar secretion a major determinant of the interaction between flowers and visitors (Willmer, 2011).

Nectaries are usually located more or less basally within the flower, with the nectar stored in spurs, concealed in corolla tubes, or enfolded (at least partially) by some other floral structures to protect against evaporation, nectar theft, fungal spores, bacteria, and/or accidental removal (due to wind, rain, or gravity); thus, the chance of anther contact would increase, owing to the inserting action the visitor must perform to reach the nectar (Bernardello, 2007; Corbet, Unwin, & Prys‐Jones, 1979; Corbet, Willmer, Beament, Unwin, & Prys‐Jones, 1979; Pacini & Nepi, 2007; Pacini, Nepi, & Vesprini, 2003; Willmer, 1983). However, fully exposed nectar can also be observed in some species with abundant pollen (e.g., certain members of Apiaceae and Asteraceae), in which case the visitors scrabble over the surface of the flower or inflorescence, spreading pollen in the process, thus leading to “mess pollination” (Corbet, 2006; Willmer, 2011).

**Figure 1:**
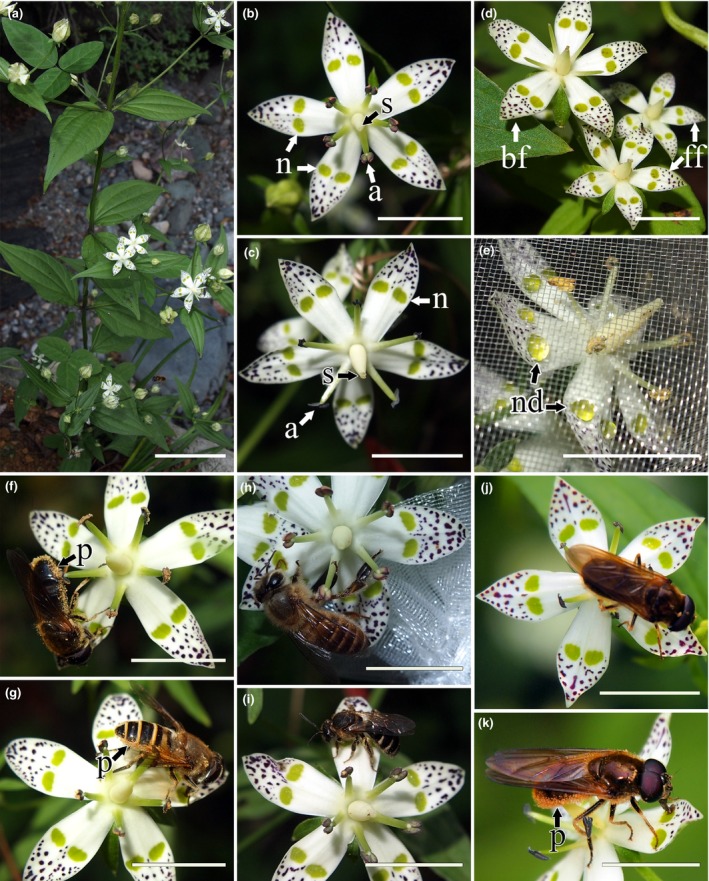
Field photographs showing floral traits and insect pollination of *Swertia bimaculata*. (a) Individual plant. (b) Monoclinous flower in the male phase. (c) Monoclinous flower in the female phase. (d) Two female flowers (lower right) compared to a monoclinous flower (top left). (e) A flower that was bagged for one day, showing nectar drops. (f, g) Two common Diptera visitor species with typical behavior, that is, crawling along the nectaries and circling on the corolla so that the insect body is constantly outside the androecium circle. (h, i) Two common Hymenoptera visitor species with the typical behavior. (j, k) A Diptera visitor with atypical behavior, which leads part of the insect's body to get into the androecium circle (j, top view; k, side view). a, anther; bf, bisexual/monoclinous flower; ff, female flower; n, nectary; nd, nectar drop; p, pollen; s, stigma. Bars = 50 mm (a) and 10 mm (b–k)

Some Gentianaceae species (i.e., some *Swertia* spp.) evolved a rather peculiar form of corolla nectaries with a flat spot‐like shape, which are located in the middle of the corolla lobe instead of at its base. These nectaries, which usually differ in color from the rest of the corolla, are arranged in a conspicuous ring on the rotate corolla, and the nectar secreted by these “gland patches” is entirely exposed (Figure 1). There is no record of the floral visitors or pollinators of these species in the literature, and hence, knowledge of their pollination ecology is limited. Gland patches are derived from primitive forms of nectaries that occur at the base of the corolla lobe and are found in most species of *Swertia* (Chassot, Nemomissa, Yuan, & Küpfer, 2001; He, Xue, & Wang, 1994). These nectaries are generally concave and have fimbriate margins (Xue, He, & Li, 2002). Little has been published on the pollination of *Swertia*. According to the literature, the most frequent visitors and primary pollinators of *Swertia* spp. with fimbriate nectaries are probably bees (Duan & Liu, 2003, 2007; Khoshoo & Tandon, 1963).

In the traditional sense, the rotate corolla of *Swertia* spp. displays a generalist pollination syndrome (Figure 1b,c), that is, plants with this corolla shape are usually pollinated by “mess pollination” by a range of fairly generalist visitors (Willmer, 2011). It is possible, though, that the characteristic gland patches play an important role in the flower–visitor interaction, influencing the preference, behavior, and/or pollination effectiveness of visitors, resulting in a relatively selective and ordered pollination pattern different from typical generalist pollination. We hypothesized that gland patches may have two possible effects on pollination: (1) visual attraction, as the yellow‐green nectaries are arranged in a conspicuous ring‐like pattern on the corolla (Figure 1b–d), which might be identified by floral visitors within a proper distance range and consequently attract more potential visitors to the flower; and (2) localization and guidance, that is, manipulation of the visitors, where the nectary whorl acts like a ring‐shaped track, urging the feeding insects to keep moving on the corolla and using nectar as a food reward.

In this study, we tested these hypotheses by investigating the pollination ecology of *Swertia bimaculata* Hook. f. & Thomson ex C. B. Clarke, a species with gland patches (Figure 1a), to understand the role that gland patches played in pollination. A comparative study was made between *S. bimaculata* and a congener, *Swertia kouitchensis* Franch., which has fimbriate nectaries, to examine morphological and functional differences between the two types of nectaries. Specifically, this study addressed the following questions: (1) How does pollination differ between species with these two types of nectaries? (2) How do gland patches affect visitor preference, behavior, and pollination effectiveness? and (3) Have other reproductive strategies associated with gland patches been developed by *S. bimaculata*?

## 2 MATERIALS AND METHODS

### 2.1 Study site

*Swertia bimaculata* is an annual plant species that is widely distributed in southern China, and its habitat elevation ranges from 250 to 3,000 m above sea level (a.s.l.). Several natural populations inhabiting Mount Guanmen, located on the eastern Ta‐pa Mountains, Shennongjia National Nature Reserve, Hubei Province, China, were used in this study. The majority of the fieldwork was carried out in a relatively large population (31°26.889′N, 110°23.422′E, 1,270 m a.s.l.) containing more than 30 individuals distributed in a vertical band on a hillside. On average, seven flowers were presented daily, and approximately 30 flowers were produced over the lifetime of each individual in the population. Both monoclinous and unisexual female flowers were observed in the population. The dominant neighboring herbs were *Artemisia* spp. (Asteraceae). A nearby *S. kouitchensis* population (approximately 100 m away) surrounded by similar plant species was used for comparisons. Our experiments with *S. bimaculata* were mainly carried out in September 2012 and September 2013, and those using *S. kouitchensis* were conducted in September 2013.

### 2.2 Sexual system

The sexual system investigation was conducted in 22 successive days (September 9–30) in 2012. All 37 individuals within the *S. bimaculata* population were labeled. On each day, the number of all monoclinous and female flowers on each individual was recorded (floral buds and postanthesis flowers were not included). The proportion of unisexual flowers among the population was calculated daily, and the mean proportion of unisexual flowers was estimated by averaging the values over 22 days.

### 2.3 Floral lifespan and morphology

The lifespan of 30 monoclinous flowers randomly selected from the population was observed. The timing of each floral event was recorded, including the opening/closure of the corolla, the beginning/ending of pollen dispersal, and the exposure/deactivation of the stigma, which was defined by the physical appearance of the stigma (e.g., coloration and texture). The duration of male, interval, and female phases were then calculated using these data.

A set of 68 flowers was randomly selected from the population to collect morphological data for floral organs, including corolla diameter, nectary whorl diameter, stamen length, androecium diameter, and pistil length. Measurements between different types of flowers (e.g., between male‐ and female‐phase monoclinous flowers) were compared. A similar investigation was conducted in *S. kouitchensis*, during which 30 randomly selected *S. kouitchensis* flowers were used for morphological measurements. Another 50 randomly selected monoclinous *S. bimaculata* flowers (including both male‐ and female‐phase monoclinous flowers) were measured to quantify positional relationships among the nectary whorl, androecium, and stigma (i.e., distances between each pair); the measurements were grouped based on floral age and sexual phase, and mean values between different groups were compared. All floral measurements were taken using a Vernier caliper and then converted to geometrical standards using the appropriate algorithm (e.g., the distance between two nonadjacent anthers in a flower was measured using the caliper and then converted to the androecium diameter using the law of sines).

### 2.4 Mating system

In total, 18 monoclinous buds were randomly collected from the *S. bimaculata* population in 2012 and 2013. The five anthers from each bud were ground in a centrifuge tube as 1 ml of water was gradually added, until the pollen grains were fully released. The tubes were well shaken, and 12–25 samples (1.5 μl each) of the pollen suspension were transferred onto a microscope slide using a pipette. Pollen grains in each sample were observed and photographed under a microscope (Olympus BX43, Tokyo, Japan). The number of pollen grains in each sample was counted from one field of view, and the number of pollen grains per flower (P) was calculated. The ovary of each bud was dissected under a stereomicroscope (Olympus SZX2, Tokyo, Japan), all ovules were photographed, and the number of ovules (O) was counted from the photograph. The pollen/ovule coefficient (P/O) for each bud was then calculated as an indication of the mating system (Cruden, 2000).

To better characterize the mating pattern of *S. bimaculata*, six treatments were established for six groups of monoclinous flowers: (A) emasculated and bagged (eight flowers from five individuals), (B) bagged only (11 flowers from two individuals), (C) emasculated and freely pollinated (eight flowers from four individuals), (D) freely pollinated flowers without any treatment (18 flowers from four individuals), (E) artificially selfed with sufficient geitonogamous pollen (10 flowers from seven individuals), and (F) artificially outcrossed with sufficient pollen from other individuals (six flowers from four individuals). Fruit and seed set of unisexual female flowers were also investigated (group G): two individuals bearing mostly female flowers were pollinated under natural circumstances, and ovaries of the female flowers were then collected. Seven to fourteen days after anthesis (before the ovaries dehisced), the ovaries from each group were collected. Each ovary was dissected under a stereomicroscope (Olympus SZX2, Tokyo, Japan) to expose all ovules, which were photographed. Using the photograph, the number of matured (M), aborted (A), and unfertilized (U) ovules in each ovary was recorded separately, and the seed‐set coefficient was calculated as M/(M + A + U). The numbers of matured and total ovules and the seed‐set coefficients between each pair of groups were compared.

In the pollen dispersal experiment, three groups of *S. bimaculata* anthers (from 22 monoclinous flowers of approximately the same size) were collected and catalogued. The control group (A) had anthers that had just dehisced; the bagged group (B) had flowers covered with gauze bags for one day after anther dehiscence, and the pollinated group (C) had flowers that were left under natural conditions for 0.5–1 day after anther dehiscence. Each anther was ground inside a centrifuge tube until the pollen grains were fully released as 1 ml of water was gradually added. The tube was well shaken, and three samples of 20 or 40 μl were transferred onto separate microscope slides using a pipette. Pollen grains in each sample were observed and counted under a microscope (Olympus BX43, Tokyo, Japan), and the number of pollen grains in each anther was estimated from the average of the three samples. Values between the three groups were compared. In this study, gauze bags were used exclusively for bagging treatments, as the material allows ventilation and effectively excludes visitors. In most cases, a single gauze bag (5 cm × 7 cm) was used for each single flower; in group B of the mating pattern experiment, two larger bags (20 cm × 30 cm) were used to cover two inflorescence branches from two individuals.

### 2.5 Nectary morphology and nectar secretion

Several buds that were about to open were collected from *S. bimaculata* and *S. kouitchensis* populations and fixed in formalin–acetic acid—70% ethanol (5:5:90, v/v). Corolla lobes were carefully detached, dehydrated in an alcohol series ranging from 50% to 95%, passed through an iso‐pentanol acetate series (SCR, Shanghai, China), and critical point dried in CO_2_ before being sputter coated with gold. The surfaces of gland patches and both sides of the corolla lobes were observed and photographed with a scanning electron microscope (SEM; S‐3400N, HITACHI, Tokyo, Japan).

A nectar secretion experiment was conducted over 10 successive days (September 14–23, 2013). On the first morning, 20 flowers randomly selected from the population were labeled and bagged and their current floral age and sexual phase were recorded separately. On the second morning (approximately 24 hr later; the specific interval was recorded for adjustment), bags were removed and the volume (V, μl) of the nectar drops on the five petals of each flower was measured with a volumetric capillary (range: 20 μl). Sugar concentration in the nectar (C, mg sugar/mg nectar) was measured with a hand refractometer and sugar content (S, mg) was then calculated as V × C, as the density of low‐concentration sugar solutions can be roughly regarded as 1 mg/μl. If nectar was obviously secreted (at least 1 μl), the bag was replaced over the flower; otherwise, another first‐day flower was randomly selected and bagged to keep a constant total number (20) of bagged flowers. This procedure was repeated daily for 10 days, but after the fifth day, no new flowers were added to the sampling set. In total, 33 flowers were recorded during the 10 days. Daily nectar volume, sugar concentration, and sugar content were compared between floral dates and between male‐ and female‐phase monoclinous flowers.

### 2.6 Insect pollination and effects of nectaries

Two experiments were established to examine the effect of gland patches on pollination, both using monoclinous flowers randomly selected from a single plant that presented a daily average of more than 30 flowers. In experiment 1, there was a control group of seven flowers (A) and a group of five flowers in which four of the five petals were removed from each flower (B). In experiment 2, there was a control group of four flowers (A), a group of four flowers in which spotted areas on the top half of the petals were covered with white paper tape, leaving the gland patches exposed (B), and a third group of five flowers where gland patches were covered with white paper tape, leaving the spotted areas exposed (C). In each experiment, flowers in different groups were observed simultaneously for 15 min under natural field conditions, and the number of insect visits to each flower was recorded along with duration of each visit. Number and duration of visits between groups were compared.

Over the two periods of data collection, the insect species visiting *S. bimaculata* flowers and their behavior were recorded through photographs and field notes. Based on observations, behaviors were categorized into several modes according to the feeding course in the flower (e.g., the landing place and the feeding order among the petals) and the way of movement between petals (e.g., flying or crawling). Visitor species were divided into several groups based on taxonomy and body size. For a more detailed analysis of pollination behavior and effectiveness, insects visiting *S. bimaculata* flowers on September 14, 15, 16, and 22 in 2013 were filmed with digital cameras fixed to tripods for 24.5 hr; during each video session, two to six flowers were captured in the screen. The total video monitoring of 21 flowers (from five individuals) added up to 101 hr. The videos were later analyzed to record the details of the visit events, including floral type (monoclinous male phase, monoclinous female phase, unisexual female, or monoclinous postfemale phase), visiting date and time, interval time for nectar secretion (Int; i.e., time elapsed between the departure of the last visitor to the arrival of the subsequent visitor; if the last visitor was still in the flower when the next visitor arrived, Int was regarded as zero), visitor species, visit duration, behavior mode, number of petals the visitor probed (P), whether anthers were touched and how many times they were touched (A), whether the stigma was touched and how many times it was touched (S), and whether insects fed on anthers and the number of times this occurred. A visit was judged effective if the anthers were touched at least once in male‐phase monoclinous flowers or if the stigma was touched at least once in female‐phase monoclinous or female flowers. The visiting frequency was calculated and compared between different insect species, pollinator groups, and floral types. Pollination stability and effectiveness at both individual and colonial levels were quantified. At the insect colonial level, for each visitor species or group, the number of effective visits per flower per hour was chosen as an index of pollination stability, and the number of times that the anthers (stigma) were (was) touched per male‐phase monoclinous flower (female‐phase monoclinous flower/female flower) per hour was chosen as an index of pollination effectiveness. At the individual insect level, for each visitor species or group, the probability of an effective visit per visit was chosen as an index of pollination stability, and the number of times that the anthers (stigma) were (was) touched in a male‐phase monoclinous flower (female‐phase monoclinous flower/female flower) per visit was chosen as an index of pollination effectiveness. These indexes between different visitor groups were compared. Several common pollinator species from the video were selected. For each selected species, at least one individual was trapped using an insect net and photographed in both dorsal and lateral views next to a scale. The photographs were analyzed using ImageJ software, and the body size of each species was measured (calculated as average thorax width and thorax height). The correlation between pollinator species’ body size and average pollination effectiveness (at the individual insect level, indicated by the average A or S of the species) was examined in male‐ and female‐phase monoclinous flowers, respectively. The correlation between pollinator species’ average A (in male‐phase monoclinous flowers) and average S (in female‐phase monoclinous flowers) was also examined. The correlations between P, Int, and A or S were examined. In *S. kouitchensis*, similar flower monitoring and video analysis were conducted. The videos lasted for 33 hr in total, and the monitoring time of all single flowers added up to 100 hr. The visiting proportions of insect groups between *S. bimaculata* and *S. kouitchensis* were compared.

### 2.7 Statistical analysis

Chi‐squared tests were used for nominal measurements: visiting frequency between floral types, pollination stability between visitor groups, and visiting proportions of insect groups between *S. bimaculata* and *S. kouitchensis*. Fisher's exact test was used if *N* < 40 or at least one cell had an expected count of less than one. Otherwise, Pearson's chi‐square test was used. For interval or ratio measurements, the data were tested for normality (using Shapiro–Wilk tests) before further statistical analysis. If normality could not be achieved after data transformation, nonparametrical tests were used: Wilcoxon signed ranks tests for paired samples, and Mann–Whitney tests and Kruskal–Wallis tests for independent samples (Wilcoxon signed ranks: daily nectar volume; sugar concentration; and sugar content between each pair of floral dates. Mann–Whitney: morphology measurements between male‐ and female‐phase monoclinous flowers; the numbers of matured and total ovules and the seed‐set coefficients between each pair of groups in the mating pattern experiment; daily nectar volume, sugar concentration, and sugar content between male‐ and female‐phase monoclinous flowers; and pollination effectiveness between visitor groups. Kruskal–Wallis: the number of matured and total ovules; and the seed‐set coefficient among groups C, D, E, and F in the mating pattern experiment). Otherwise, parametrical tests were used to compare mean values between groups: independent sample *t* tests for two groups and one‐way ANOVA for multiple groups (two‐sample *t* test: positional relationship of nectary whorl, androecium, and stigma between male‐ and female‐phase monoclinous flowers and between each pair of floral dates; and number and duration of visits between groups in nectary effect experiment 1. ANOVA: the number of pollen grains between groups in the pollen dispersal experiment; and number and duration of visits between groups in nectary effect experiment 2. The natural logarithm of visit duration was used so that the data had a normal distribution.). Tukey's HSD and Dunnett's *t* were used as post hoc tests after ANOVA. Bivariate correlation and linear regression were used to examine the relationship between two interval or ratio variables (P‐A, P‐S, P‐Int, Int‐A, and Int‐S in the video analysis). If the data did not conform to a normal distribution, Spearman's ρ was used as the correlation coefficient; otherwise, Pearson correlation coefficients (*r*) were used. Values are presented as means ± standard deviation (*SD*) in the results, unless stated otherwise.

## 3 RESULTS

### 3.1 Sexual system

Both monoclinous and female (i.e., male sterile) flowers were observed in *Swertia bimaculata* populations. Stamens of the latter flower type were usually absent, although a minority had rudimentary and infertile stamens (Figure 1d). The proportion of female flowers in the experimental population averaged 9.7% ± 3.3%.

### 3.2 Floral lifespan and morphology

Once the corolla opened, anthers of the monoclinous flowers dehisced in 0.9 ± 0.8 hr (*N* = 19). When anthers dehisced and pollen began to disperse, the flower was considered to be in the male phase, which lasted for 0.5 ± 0.5 days (*N* = 14) under natural conditions, until all pollen inside the anthers was dispersed. The male phase was followed by the interval phase, when the stigma was not yet exposed, and this phase lasted for 1.0 ± 0.4 days (*N* = 15). The flower entered the female phase as soon as the stigma was exposed (two stigma lobes opened, exposing the receptive surface) and could receive pollen, which lasted for 2.1 ± 0.7 days (*N* = 9). The corolla then gradually closed over the next 2.0 ± 0.8 days (*N* = 10). As the rate of pollen dispersal varied with insect visiting frequency, the duration of the male phase was strongly influenced by external factors (e.g., weather); this was noted in field observations. Therefore, the phase between anther dehiscence and stigma exposure was considered a generalized “male phase” in this study. The duration of this generalized male phase was stable, as it was not substantially influenced by external factors. The division of sexual phases does not apply to female flowers because they have no fertile male organs. Hereafter, “male‐phase” or “female‐phase” flowers refer to monoclinous flowers in their generalized male or female phase, unless stated otherwise.

In *S. bimaculata*, female flowers had significantly smaller diameters of corolla, nectary whorl, and androecium than monoclinous flowers, and their pistil lengths were shorter (Table 1; Figure 1d). Androecium diameter was significantly larger in female‐ than in male‐phase flowers (*p* < .001), indicating an obvious stamen movement away from the stigma after anther dehiscence in monoclinous flowers (Table 1; Figure 1b,c). In addition, during anthesis, *S. bimaculata* nectaries were always more distant from the flower axis than the androecium was (Table 1; Figure 1b,c), contrary to what was found in *S. kouitchensis* (Table 1). These results indicated that the floral morphology in *S. bimaculata* and *S. kouitchensis* was quite different, especially as reflected in the positional relationship between nectaries and other floral structures.

**Table 1:** Floral organ measurements in *Swertia bimaculata* and *S. kouitchensis*

	*S. bimaculata*			*S. kouitchensis*
	Monoclinous		Female	
	Male phase^a^	Female phase		
Corolla diameter	24.7 ± 3.7 (31)	26.9 ± 3.4 (24)	14.8 ± 2.0 (10)	15.8 ± 1.3 (29)
Nectary whorl diameter	13.4 ± 2.2 (31)	14.9 ± 1.9 (24)	8.0 ± 1.2 (10)	3.2 ± 0.2 (29)
Androecium diameter	8.2 ± 2.3 (31)	12.5 ± 1.6 (24)	0.4 ± 1.1 (10)^b^	3.6 ± 0.6 (29)
Stamen length	5.3 ± 0.5 (31)	5.9 ± 0.5 (24)	0.1 ± 0.3 (10)^b^	4.2 ± 0.3 (29)
Pistil length	5.6 ± 1.1 (31)	6.5 ± 1.0 (24)	4.3 ± 0.8 (10)	4.2 ± 0.4 (29)

Mean values ± *SD* are displayed, and sampling size (*N*) is shown in parentheses.

a Includes the interval phase.

b In the 10 female flowers, only one had vestigial stamens that could be measured, and data for the others were recorded as zero.All measurements are in millimeters (mm).

**Figure 2:**
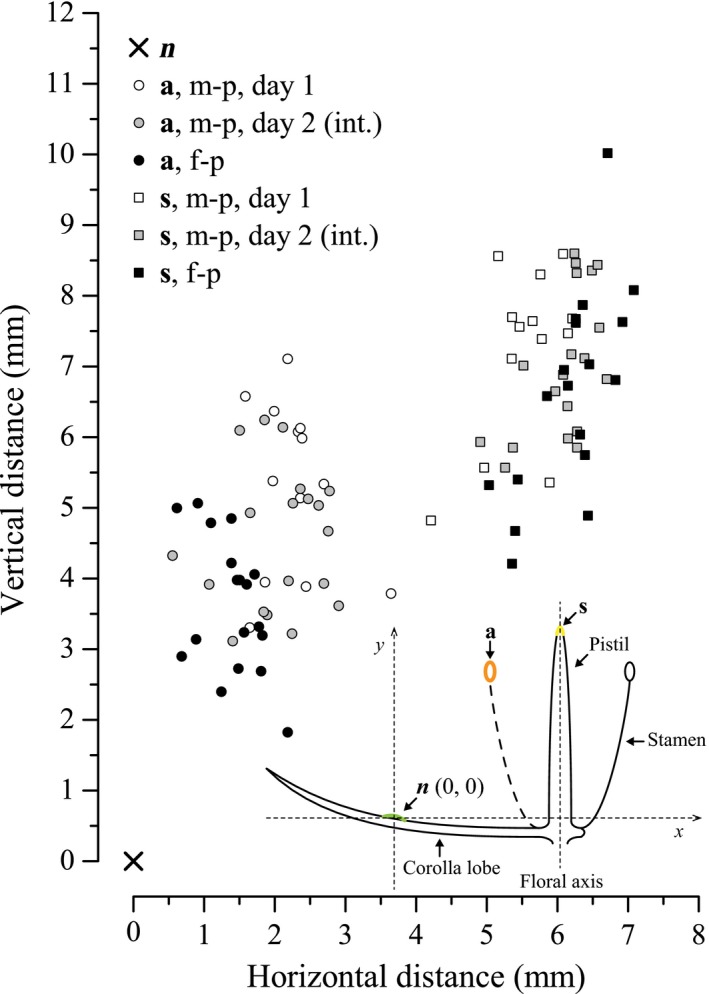
Positional relationships between nectaries, anthers, and stigmas in male‐phase, interval, and female‐phase *Swertia bimaculata* flowers. Fifty monoclinous flowers were measured. For each flower, an anatomically longitudinal section that passed through the flower axis and a nectary was chosen and fixed at coordinates (0, 0). The anatomically horizontal/vertical direction was then defined as the *X*/*Y*‐axis, and the coordinates of the stigma were recorded. Because stamens alternated with corolla lobes, the section plane can only pass through an anther on the opposite side of the floral axis. The symmetry point of this anther with respect to the floral axis was selected as the coordinate of the “anther”. Thus, the nectary–anther distance in this figure is exactly equal to the shortest distance from the nectary whorl to the androecial circle (see the diagrammatic sketch in the figure, which shows the longitudinal section of a flower). a, anther; f‐p, female phase; int., interval; m‐p, male phase; n, nectary; s, stigma

As the flower aged, anthers (**a**) clearly moved away from the stigma (**s**), that is, the distance from **a** to **s** significantly increased (male‐ to interval phase: *t* = 3.49, *df* = 30, *p* = .002; interval‐ to female phase: *t* = 3.34, *df* = 35, *p* = .002), which would lead to distinct herkogamy at the beginning of the female phase (Figure 2; Table 2). Additionally, the distance from **n** (nectary) to **a** was significantly reduced (male‐ to interval phase: *t* = 2.09, *df* = 30, *p* = .045; interval‐ to female phase: *t* = 3.85, *df* = 35, *p* < .001), whereas the distance from **n** to **s** did not change significantly (male‐ to interval phase: *t* = 0.47, *df* = 30, *p* = .64; interval‐ to female phase: *t* = 0.52, *df* = 35, *p* = .61; Figure 2; Table 2).

**Table 2:** Relative distances between nectary (n), anther (a), and stigma (s) in *Swertia bimaculata*

	s–a	n–a	n–s
Male phase, day 1	3.88 ± 0.67 (13)^a^	6.30 ± 0.90 (13)^a^	9.11 ± 1.17 (13)^a^
Male phase, day 2 (interval)	4.76 ± 0.72 (19)^b^	5.50 ± 0.85 (19)^b^	9.29 ± 0.99 (19)^a^
Female phase	5.70 ± 0.97 (18)^c^	4.54 ± 1.93 (18)^c^	9.09 ± 1.36 (18)^a^

Mean values ± *SD* are displayed and sampling size (*N*) is shown in parentheses. For each column, different superscript letters indicate statistically significant difference at α = 0.05 (two‐sample *t* test).

### 3.3 Mating system

The number of pollen grains per monoclinous flower (P) averaged 62 406 ± 12 155 (*N* = 8) in 2012, whereas the number of ovules per flower (O) averaged 73.3 ± 18.8; thus, P/O ranged between 721 and 1,123 and averaged 870 ± 148. In 2013, P averaged 10,5514 ± 17,486 (*N* = 10), whereas O averaged 112.9 ± 11.1, and P/O varied from 588 to 1,156 with an average of 945 ± 188. The P and O values differed significantly between the 2 years (*t* = 5.91, *df* = 16, *p* < .001; *t* = 5.58, *df* = 16, *p* < .001), but the P/O ratio did not (*t* = 0.92, *df* = 16, *p* = .37). The average P/O over the 2 years was 912 ± 171 (*N* = 18), indicating that the *S. bimaculata* mating system is facultatively xenogamous, according to Cruden (2000).

In the mating pattern experiment, fruit‐set coefficients, seed numbers per capsule, and seed‐set coefficients of the six groups of monoclinous flowers were obtained (Table 3). None of the flowers in group A (bagged and emasculated) set fruits, suggesting that apomixis did not occur. A minority within group B (bagged flowers without emasculation) set fruits with insufficient seed‐set rates.

**Table 3:** Fruit and seed set in *Swertia bimaculata* monoclinous flowers under six pollination treatments (A–F) and in female flowers under natural conditions (G)

	Fruit‐set rate (%)	Matured ovules (number)	Seed‐set rate (%)
A: Bagged and emasculated	0.0 (0 of 8)	0 ± 0^a^	0.0 ± 0.0^a^
B: Bagged	36.4 (4 of 11)	16 ± 27^a^	20.3 ± 32.7^a^
C: Emasculated and freely pollinated	100.0 (8 of 8)	93 ± 24^b^	82.2 ± 15.7^b^
D: Freely pollinated	94.4 (17 of 18)	89 ± 31^b^	83.1 ± 22.6^b^
E: Artificially selfed	100.0 (10 of 10)	76 ± 33^b^	83.8 ± 27.7^b^
F: Artificially outcrossed	100.0 (6 of 6)	89 ± 44^b^	77.0 ± 17.8^b^
G: Freely pollinated female flowers	100.0 (14 of 14)	44 ± 10^c^	94.3 ± 8.3^c^

The number of matured ovaries and sampling size are given in parentheses. Error is presented as ± *SD*. For each column, different superscript letters indicate a statistically significant difference at α = 0.05 (Mann–Whitney test).

All flowers in group C (emasculated and freely pollinated) and nearly all flowers in group D (freely pollinated without emasculation) set fruits. Neither the number of matured ovules (*Z* = −0.36, *p* = .72) nor the seed‐set coefficient (*Z* = −0.83, *p* = .41) differed significantly between these two groups, indicating that emasculation had no significant influence on the pollination of *S. bimaculata* and that monoclinous flowers could achieve sufficient seed set through allogamy. All flowers in groups E (selfed) and F (outcrossed) set fruits and there was no significant difference in either the number of matured ovules (*Z* = −1.06, *p* = .29) or the seed‐set coefficient (*Z* = −0.86, *p* = .41) between the two groups, suggesting that *S. bimaculata* is self‐compatible. The number of matured ovules (chi‐squared = 2.00, *df* = 3, *p* = .57) and the seed‐set coefficient (chi‐squared = 3.57, *df* = 3, *p* = .31) did not differ significantly among groups C, D, E, and F, suggesting there was no noticeable pollen limitation in the natural population.

All freely pollinated female flowers (group G) set fruits, and their seed‐set coefficient was significantly higher than that of freely pollinated monoclinous flowers (group D) (*Z* = −2.89, *p* = .004), although the number of matured ovules per female flower was significantly lower owing to a lower ovule abundance.

**Figure 3:**
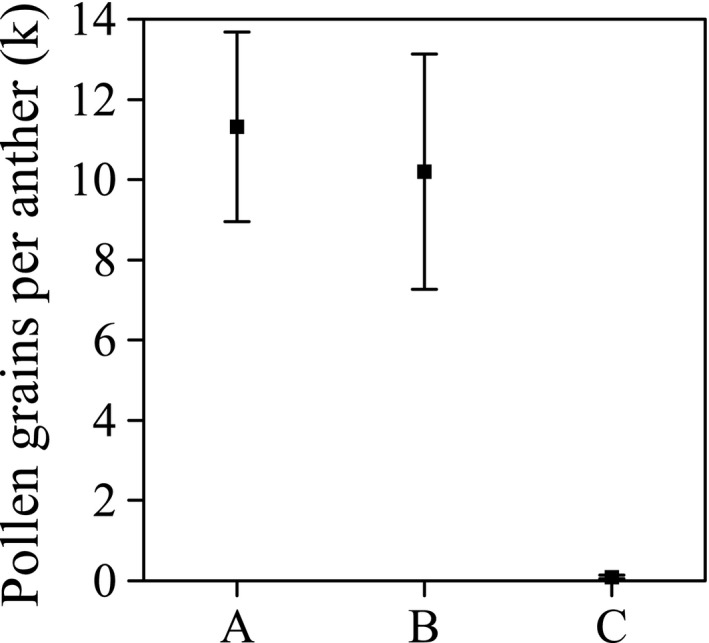
Pollen dispersal in flowers whose pollinators were excluded compared to flowers that were freely pollinated. Mean values (±*SD*) of three groups of anthers are shown: (a) just dehisced, (b) 1 day after dehiscence, flowers bagged, and (c) 0.5–1.0 day after dehiscence without bagging the flowers

In the pollen dispersal experiment, there was a significant difference among the three groups of anthers (A, just dehisced control; B, bagged for 1 days; and C, left under natural circumstances for 0.5–1.0 day; *F*_2,16_ = 49.53, *p* < .001), and the post hoc test revealed no significant difference between groups A and B (*p* = .574) but a significant difference between groups A and C (*p* < .001). The results indicated that, when insect pollinators were present, little pollen was left on stamens after 0.5–1 days of free pollination, reflecting the rapid and thorough entomophilous pollen dispersal. However, when visitors were excluded from the flowers by gauze bags, pollen grain number per anther did not decrease significantly after 24 hr, suggesting that wind and gravity exert little influence on *S. bimaculata* pollination (Figure 3).

### 3.4 Nectary morphology and nectar secretion

**Figure 4:**
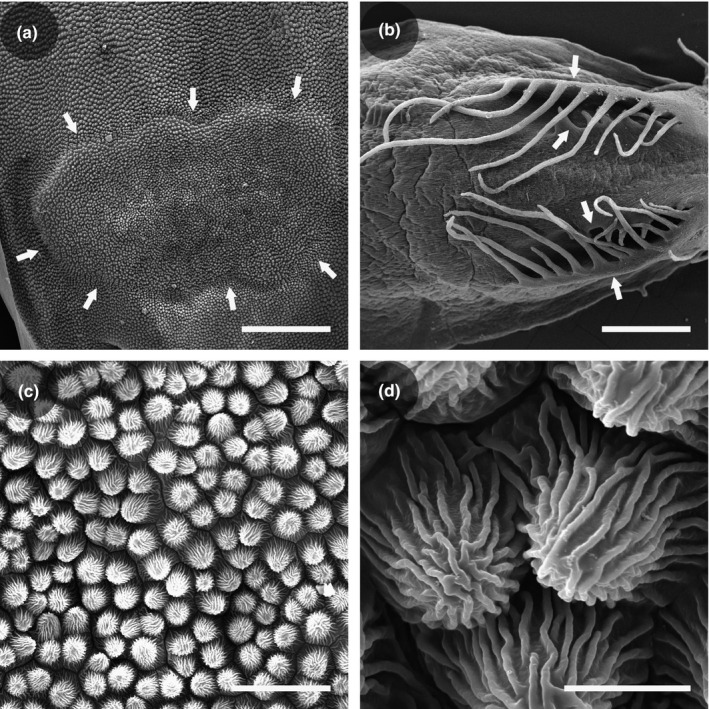
Nectaries of *Swertia bimaculata* and *Swertia kouitchensis* imaged by scanning electron microscopy. (a) Full view of a *S. bimaculata* gland patch, with arrows indicating the border of the nectary. (b) Fimbriate nectaries on a lobe of a *S. kouitchensis* corolla, with arrows indicating the margins of each nectary. (c) Partial view of a *S. bimaculata* gland patch, showing the array of papillose epidermal cells. (d) Close‐up of the epidermal cells in a *S. bimaculata* gland patch. Bars = 500 μm (a and b), 50 μm (c), and 10 μm (d)

Gland patches were observed as flat structures that slightly bulged out from the corolla. Adaxial epidermal cells of the corolla had similar shapes inside and outside the gland patch area (Figure 4a). These papillose cells were compactly arranged in intervals approximately 10–20 μm between two neighboring cells along the upper corolla surface (Figure 4b,c). Such microrelief was not found in *S. kouitchensis* flowers (Figure 4d).

**Figure 5:**
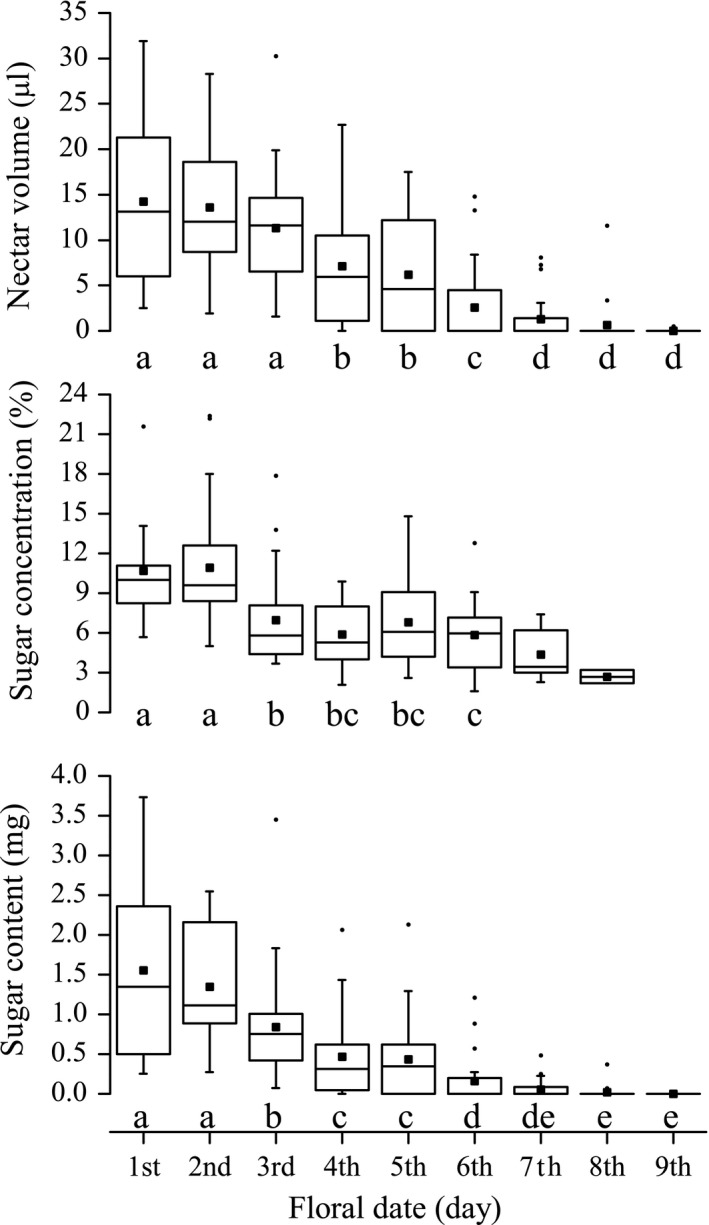
Temporal pattern of nectar secretion during anthesis in *Swertia bimaculata*. Daily nectar volume (i.e., nectar standing crop), sugar concentration, and sugar content in the nectar of each flower are shown in box‐and‐whisker plots. The bottom/top of the box indicates the first/third quartile, and the band inside the box indicates the second quartile (i.e., the median). The lower/upper whisker ends at the lowest/highest datum within the 1.5 IQR (interquartile range) of the lower/upper quartile; outliers are marked using dots collinear with the whiskers. Mean values are indicated with blocks inside the boxes. In each coordinate system, different annotation letters indicate a statistically significant difference at α = 0.05 (Wilcoxon signed ranks test)

**Figure 6:**
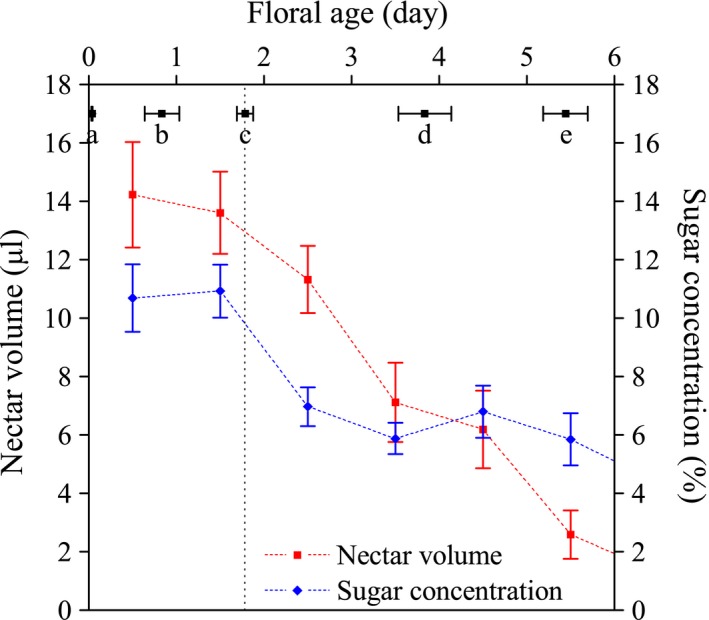
Comparison between floral lifespan and nectar secretion in *Swertia bimaculata*. The anthesis floral events are indicated as (a) anther dehiscence, (b) pollen dispersal end, (c) stigma exposure, (d) stigma deactivation (judged by color and texture), and (e) corolla closure. Mean values of nectar volume and sugar concentration are graphically represented by red and blue marks, respectively. Error bars indicate standard error (*SE*). The vertical dotted line delimits male and female phases

Nectar secretion of a monoclinous flower lasted for 3–8 days (4.9 ± 1.6 days, *N* = 23), during which a total of 14.4–124.7 μl (57.2 ± 29.1 μl, *N* = 19) of nectar was secreted (Figure 5). The average daily nectar volume before stigma exposure, that is, in the first 2 days of anthesis, was significantly higher than that after stigma exposure (before: 13.9 ± 8.0 μl, *N* = 52; after: 8.5 ± 6.2 μl, *N* = 89; *Z* = −3.93, *p* < .001), indicating a highly significant difference in the rate (volume per unit time) of nectar production between male and female phases (Figure 6).

Sugar concentration in *S. bimaculata* nectar ranged from 1.6% to 22.4% (7.6 ± 4.1%, *N* = 122). It averaged 10.8 ± 4.2% (*N* = 37) in the first 2 days of anthesis and 6.2 ± 3.1% (*N* = 85) in the following days, indicating a highly significant difference in sugar concentration between the male‐ and female‐phase nectar (*Z* = −6.06, *p* < .001) (Figure 5). In addition, the first and only significant decline of daily sugar concentration occurred at the beginning of the female phase: sugar concentration averaged 10.9 ± 4.5% (*N* = 25) in the second day of anthesis and 7.0 ± 3.5% (*N* = 27) in the third day of anthesis (*Z* = −3.01, *p* = .003; Figures 5 and 6).

Daily sugar content was calculated using nectar volumes and sugar concentrations. A monoclinous flower produced 1.94–10.88 mg (4.97 ± 2.61 mg, *N* = 10) of nectar sugar during its lifespan. Its daily sugar output averaged 0.84 ± 0.77 mg (*N* = 121) throughout anthesis, and 1.41 ± 0.88 mg (*N* = 37) and 0.59 ± 0.57 mg (*N* = 84) in the male and female phases, respectively, showing a highly significant difference (*Z* = −5.52, *p* < .001) between the two phases (Figure 5). Similar to sugar concentration, the first significant decline of daily sugar content also occurred on the third day of anthesis (from 1.35 ± 0.71 mg (*N* = 25) on the second day to 0.84 ± 0.67 mg (*N* = 27) on the third day; *Z* = −2.68, *p* = .007; Figures 5 and 6). Based on these results, the daily abundance of food reward for pollinators produced by *S. bimaculata* monoclinous flowers was significantly higher (on average) in the male than in the female phase. Moreover, the daily abundance of the food reward showed a significant decline at the transition from the male to the female phase. It is noteworthy that all these changes were driven by internal mechanisms: The flowers used in the experiment were covered with gauze bags, and insects could not reach their nectaries, anthers, or stigmas.

### 3.5 Insect pollination and effects of nectaries

**Figure 7:**
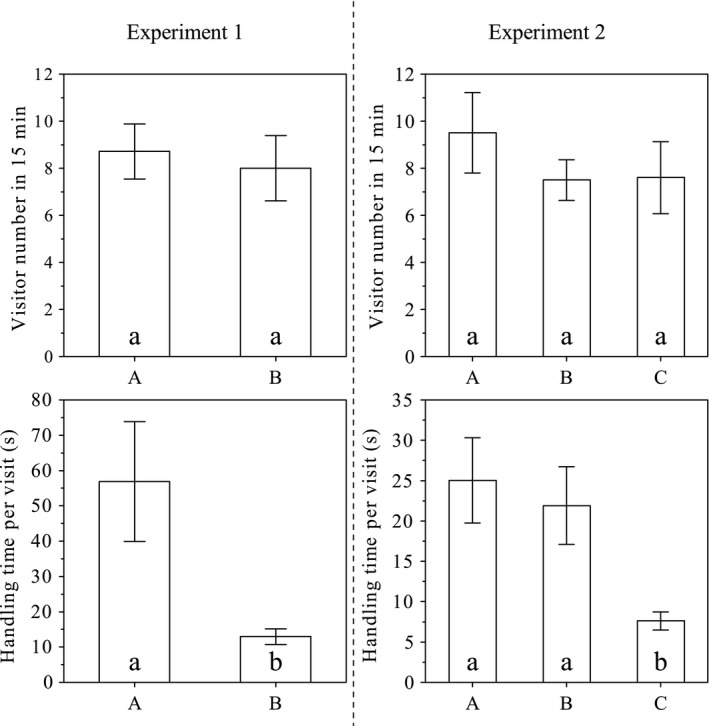
Effects of *Swertia bimaculata* nectaries on the visiting frequency and duration of insect visitors. Experiment 1: group A, control; group B, four of the five petals were removed from each flower. Experiment 2: group A, control; group B, spotted areas were covered; group C, gland patches were covered. Mean values and standard error (*SE*) are indicated by columns and error bars, respectively. In each panel, different annotation letters indicate a statistically significant difference at α = 0.05 (two‐sample *t* test)

In nectary effect experiment 1, each control flower (group A) had 6–15 (8.7 ± 3.1, *N* = 7) insect visitors in 15 min; in the same time interval, 4–12 (8.0 ± 3.1, *N* = 5) insects visited a flower in which four of the five petals were removed (group B). No significant difference was found between these two groups (*t* = 0.39, *df* = 10, *p* = .70; Figure 7a), but the time spent by a visitor on a flower averaged 56.8 ± 12.9 s (±*SE*;* N* = 56) and 17.0 ± 2.3 s (±*SE*;* N* = 40) in groups A and B, respectively, and there was a highly significant difference between these groups (*t* = 3.80, *df* = 94, *p* < .001; Figure 7b).

In nectary effect experiment 2, each flower in groups A (control), B (spotted areas covered), and C (gland patches covered) was visited by 5–13 (9.5 ± 3.4, *N* = 4), 6–10 (7.5 ± 1.7, *N* = 4), and 5–13 (7.6 ± 3.4, *N* = 5) insects in 15 min, respectively. There was no significant difference between these three groups (*F*_2,10_ = 0.58, *p* = .58; Figure 7c). The visit duration averaged 25.0 ± 5.3 s (±*SE*;* N* = 38), 21.9 ± 4.8 s (±*SE*;* N* = 30), and 7.6 ± 1.1 s (±*SE*;* N* = 38) in groups A, B, and C, respectively, and a highly significant difference among these three groups was found (*F*_2,103_ = 11.724, *p* < .001; Figure 7d). Tukey's HSD indicated that the difference was not significant between groups A and B (*p* = 1.000), but it was significant between groups A and C (*p* < .001) and between groups B and C (*p* < .001). Dunnett's *t* test indicated no significant difference between the control group (A) and group B (*p* = .999) and a significant difference between the control group and group C (*p* < .001).

Gland patches and spotted areas were the two main floral factors manipulated in nectary effect experiments 1 and 2, respectively, by either minimizing the two factors together or eliminating them separately. None of these manipulations caused a reduction in insect visiting frequency to single flowers, suggesting that neither gland patches nor spotted areas are essential to visitor attraction for a single flower. However, not all flowers in the population were manipulated; insects might be initially attracted to the nearby nontreated flowers by olfactory signals released from their nectar (long‐range attraction effect). Nonetheless, removal of both factors and blocking gland patches alone significantly decreased insect visit duration, probably owing to the lack of nectar rewards, indicating that nectaries play a crucial role in visitors’ feeding and movement on the flowers.

Field observations showed a large variety of insect visitors in the *S. bimaculata* population, most of which foraged for nectar instead of landing randomly on flowers. More than 35 insect species were recorded, including more than 26 dipteran and five hymenopteran species. Hemiptera, Lepidoptera, and Coleoptera, as well as other taxa, were also occasionally recorded. The most common bee visitors were Halictidae, Apidae, and Vespidae, whereas Syrphidae, Tachinidae, Calliphoridae, and Muscidae were the most frequent fly visitors (Figure 1f–k). In *S. kouitchensis*, however, only 13 species of insects visited flowers, including approximately five dipteran and six hymenopteran species.

Approximately 56% (649) of the 1,168 total insect visits to flowers were by bees (Hymenoptera) and approximately 42% (492) were by flies (Diptera). It should be noted that the actual proportion of fly visits in *S. bimaculata* was higher than 42% because some tiny flies were omitted during the recording owing to their irregular behavior and extremely low probability of touching either the anthers or the stigmas. A similar investigation in *S. kouitchensis* indicated that 76% of the visits (222 of 292) were by bees and only 23% (66 of 292) were by flies. The ratio of flies to all visitors was significantly higher in *S. bimaculata* than in *S. kouitchensis* (*p* < .001).

Nearly all visitors performed a “circling” behavior when feeding on *S. bimaculata* flowers, for example, searching along the nectaries successively to collect nectar, moving clockwise or anticlockwise on the corolla. When doing so, most visitor species crawled between petals, whereas others (mainly some Syrphidae spp.) flew from one petal to another. The former behavior was defined as the “typical” behavior for *S. bimaculata* pollinators because it was far more common and more likely to generate contact between the insect's body and the anthers (Figure 1f–i). Furthermore, a visitor was designated “large” if the species had this typical behavior and the individual's body length was greater than 10 mm.

The circling behavior of insects was also observed in *S. kouitchensis*, which has fimbriate nectaries. The main difference of insect behavior observed between *S. bimaculata* and *S. kouitchensis* is that in *S. kouitchensis*, the radial distance from nectary to pistil is short, allowing a pollinator to contact the stigma as well as the anthers when circling along the nectaries, whereas the long nectary–pistil distance in *S. bimaculata* flowers makes it almost impossible for a typically behaved pollinator to come in contact with the stigma (Figure 1f–i). In other words, a pollinator can contact the stigma in an *S. bimaculata* flower only by behaving somewhat “atypically.” Such behaviors mainly include (1) landing on the center of the flower near the pistil (such that the ventral/lateral part of the body can touch the stigma) rather than on the nectary ring outside androecium (Figure 1j,k), (2) moving from a petal to a nonadjacent petal instead of an adjacent petal such that one side of the body can touch the stigma, and (3) leaving the back part of the body inside the androecium such that the hind feet might contact the stigma. Video analysis showed a significantly higher probability that a large fly visiting a female‐phase flower would behave atypically compared to a large bee (flies: 39 of 62, 62.9%; bees: 16 of 218, 7.3%; *p* < 10^−21^). It is noteworthy that although “atypical” cases were observed in most of the large fly visits, circling behavior still often occurred during these visits, for example, an insect can circle on the corolla before/after moving to a nonadjacent petal.

The videos recorded 647, 455, and 40 visiting events on male‐phase flowers, female‐phase flowers, and female flowers, respectively. The monitoring time of these three types of flowers was 30.25 hr, 34.38 hr, and 9.50 hr, respectively. If there was no significant difference of insect attraction between the three types of flowers, the expected visiting frequency would be directly proportional to the monitoring time. This null hypothesis was rejected; our results indicated a significant pollinator preference among the three types of flowers (*p* < .001) and between each pair of flower types (*p* < .001). According to the observed visiting frequencies, the most attractive types of flowers for the visitors were male‐phase flowers, followed by female‐phase flowers. The female flowers had significantly fewer visitors than the monoclinous flowers (Table 4). Furthermore, in each type of flower, visiting frequency was not substantially different between bees and flies (Table 4). In all three types of flowers, the colonial stability (indicated by the number of effective visits per flower per hour) of flies was higher than that of bees, as was the colonial effectiveness (indicated by the number of times the anthers or stigma was touched per flower per hour). It is noteworthy that large flies contributed to more than 40% of the anther contacts and more than 70% of the stigma contacts in monoclinous flowers, although they only accounted for approximately 12% of all insect visitors (Table 4). Nevertheless, no large flies were recorded visiting female flowers and only a few were registered in field observations. Incidentally, pollen‐eating behavior only happened four times among the 1,168 visits, always by bees.

**Table 4:** Visiting frequency and pollination effectiveness of *Swertia bimaculata* visitors at the insect colonial level

	Monoclinous flowers						Female flowers		
	Male phase			Female phase					
	V	E	A	V	E	S	V	E	S
All insects	21.4	8.0	19.6	13.2	1.86	2.53	4.2	0.32	0.32
Bees	**11.8**	3.4	6.8	**7.4**	0.35	0.38	**3.1**	0.11	0.11
Large bees	**9.3**	**3.2**	6.6	**6.3**	0.35	0.38	**2.8**	**0.11**	**0.11**
Flies	9.0	**4.1**	**11.5**	5.6	**1.48**	**2.12**	1.2	**0.21**	**0.21**
Large flies	2.3	2.2	**8.0**	1.8	**1.19**	**1.80**	0.0	0.00	0.00

All data have units of value flower^−1^ hr^−1^. For each visitor group and flower type, the number of visits per flower per hour (V), the number of effective visits per flower per hour (E), and the number of times the anthers/stigma were/was touched per flower per hour (A or S) are given. A visit was judged effective if the anthers were touched at least once in male‐phase flowers or if the stigma was touched at least once in female‐phase or female flowers. In each column, the larger values obtained between bees and flies and between large bees and large flies are displayed in bold.

In *S. kouitchensis*, visiting frequency was 2.9 times flower^−1^ hr^−1^, which was less than that for *S. bimaculata* (17.1 times flower^−1^ hr^−1^), and the visiting frequency of bees (2.2 times flower^−1^ hr^−1^) was higher than that of flies (0.7 times flower^−1^ hr^−1^). The proportion of fly visitors to *S. bimaculata* was significantly higher than that to *S. kouitchensis* (*p* < .001).

A significantly higher individual pollination stability (indicated by the probability of an effective visit) in flies than in bees was revealed in both male‐phase flowers (bees: 102/356, flies: 124/270, *p* < .001) and female‐phase flowers (bees: 12/237, flies: 51/189, *p* < .001). In female flowers, no significant difference was found (bees: 1/24, flies: 2/11, *p* = .227), which was possibly due to the small sampling size (Table 5). A significantly greater individual pollination effectiveness (indicated by the number of times that the anthers/stigma were/was touched per visit) of flies than of bees was revealed in both male‐phase flowers (*Z* = −5.11, *p* < .001) and female‐phase flowers (*Z* = −6.39, *p* < .001). Such a significant difference was not found in female flowers (*Z* = −1.36, *p* = .175), possibly due to the small sampling size (Table 5). Furthermore, large flies had a significantly greater individual pollination effectiveness than either other flies (in male‐phase flowers, *Z* = −11.16, *p* < .001; in female‐phase flowers, *Z* = −8.535, *p* < .001) or large bees (in male‐phase flowers, *Z* = −10.85, *p* < .001; in female‐phase flowers, *Z* = −10.39, *p* < .001; Table 5).

**Table 5:** Pollination effectiveness of *Swertia bimaculata* visitors at the individual insect level

	Monoclinous flowers				Female flowers	
	Male phase		Female phase			
	E	A	E	S	E	S
All insects	0.37 ± 0.02 (644)	0.92 ± 0.06 (644)	0.147 ± 0.017 (435)	0.200 ± 0.028 (435)	0.086 ± 0.048 (35)	0.086 ± 0.048 (35)
Bees	0.29 ± 0.02 (356)	0.58 ± 0.06 (356)	0.051 ± 0.014 (237)	0.055 ± 0.016 (237)	0.042 ± 0.042 (24)	0.042 ± 0.042 (24)
Large bees	0.34 ± 0.03 (279)	0.71 ± 0.07 (279)	0.060 ± 0.017 (200)	0.065 ± 0.019 (200)	0.045 ± 0.045 (22)	0.045 ± 0.045 (22)
Flies	**0.46 ± 0.03 (270)**	**1.29 ± 0.11 (270)**	**0.270 ± 0.032 (189)**	**0.386 ± 0.057 (189)**	**0.182 ± 0.122 (11)**	**0.182 ± 0.122 (11)**
Large flies	**0.94 ± 0.03 (71)**	**3.41 ± 0.24 (71)**	**0.661 ± 0.061 (62)**	**1.000 ± 0.136 (62)**	—	—

All data have units of value per visit. For each visitor group and flower type, the mean value of the number of effective visits per visit (E) and the number of times that the anthers/stigma were/was touched per visit (A or S) are shown. E can also be regarded as the probability of an effective visit. A visit was judged effective if the anthers were touched at least once in male‐phase flowers or if the stigma was touched at least once in female‐phase or female flowers. Standard error (*SE*) is displayed after the mean values, and sampling size (*N*) is shown in parentheses. In each column, the larger values obtained between bees and flies and between large bees and large flies are displayed in bold.

**Figure 8:**
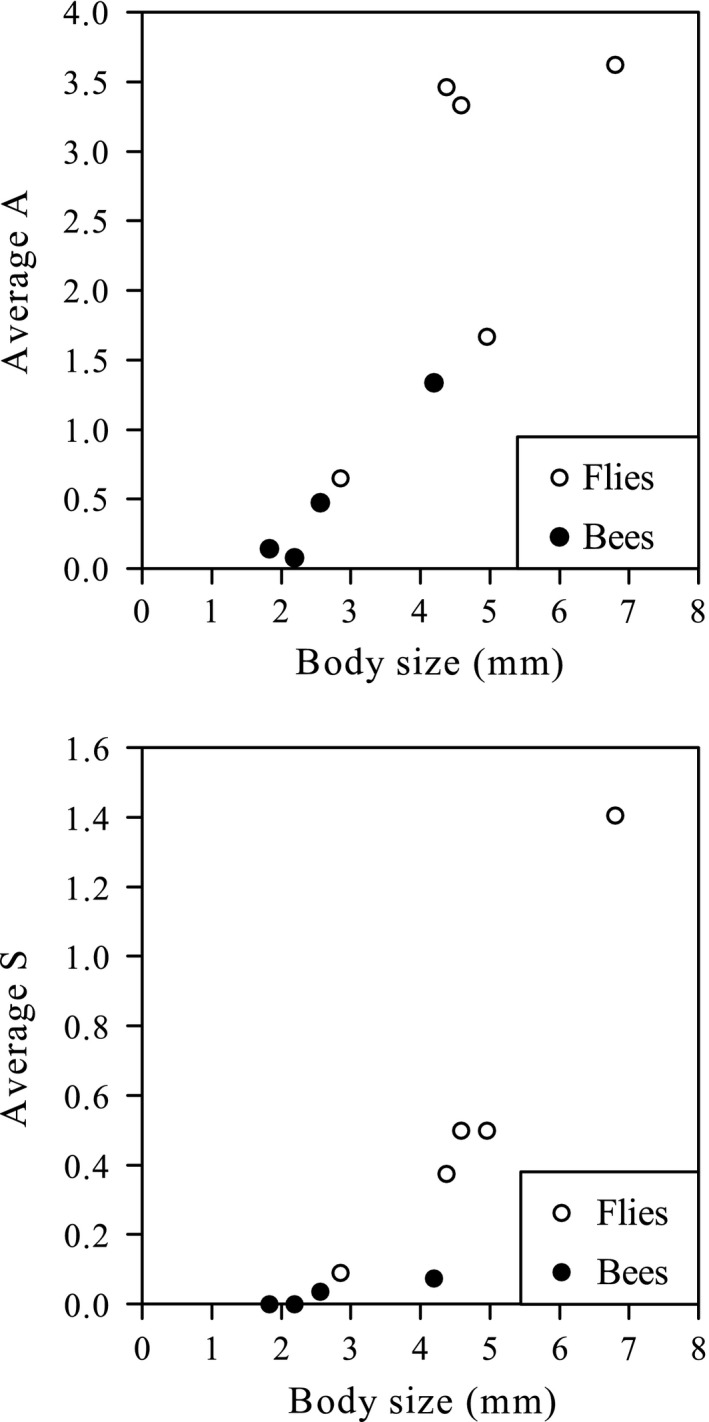
Correlation between *Swertia bimaculata* pollinator body size and A or S (number of times that the anthers/stigma were/was touched per visit). Body sizes and mean values of A or S are shown for nine common pollinator species. Male‐phase and female‐phase flower data were used separately to examine the correlation between insect body size and pollination effectiveness. The body size of each species was calculated as average thorax width and thorax height

The correlation between pollinator species’ body size and the species’ average pollination effectiveness was significant in both male‐ and female‐phase flowers (male phase: *r* = .860, *N* = 9, *p* < .01; female phase: Spearman's ρ = 0.975, *N* = 9, *p* < .01). In general, a pollinator species with a larger body size was individually more effective in both male‐ and female‐phase flowers (Figure 8). Pollinator species with higher individual pollination effectiveness in male‐phase flowers (indicated by the species’ average A) were also more likely to have higher individual pollination effectiveness in female‐phase flowers (indicated by the species’ average S). The correlation between pollinator species’ average A and S was significant (Spearman's ρ = 0.725, *N* = 15, *p* < .01). Moreover, large fly species had higher individual pollination effectiveness than bee species with a similar body size, especially in female‐phase flowers (Figure 8).

**Figure 9:**
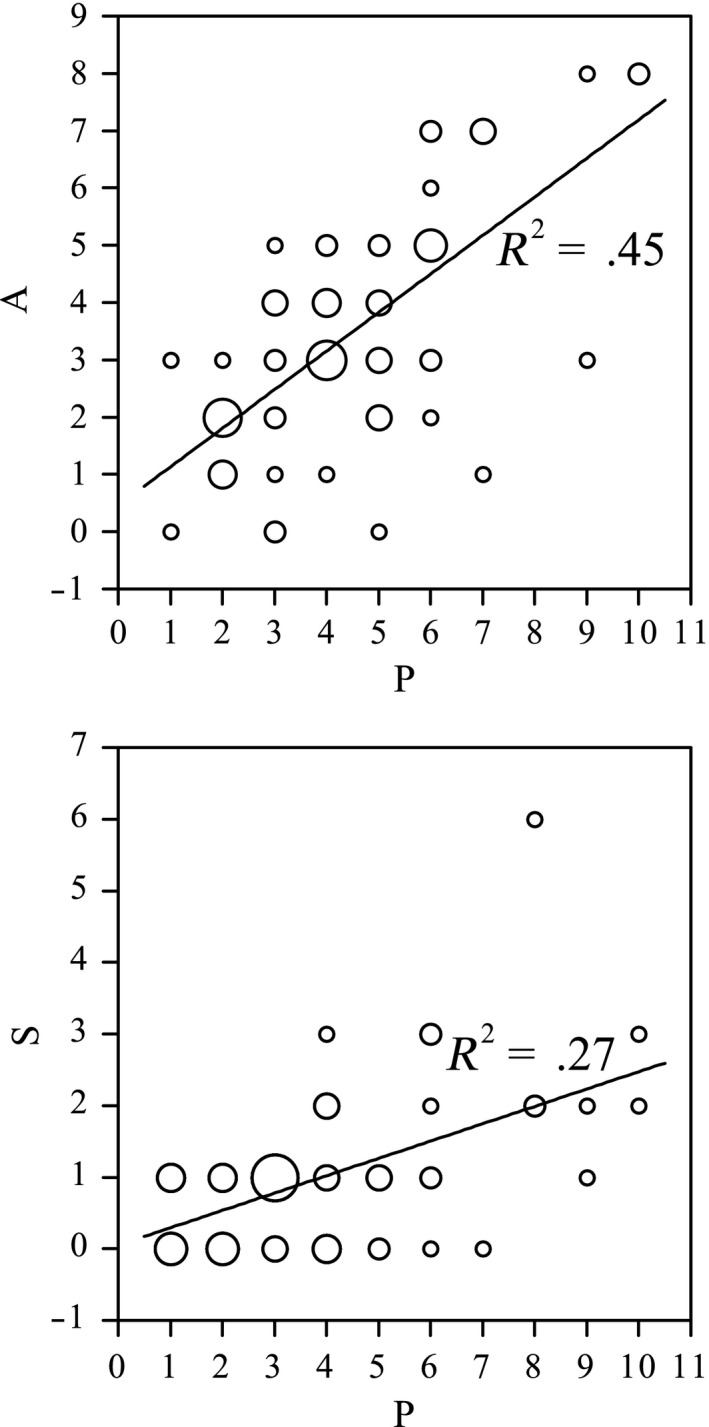
Correlation between P (number of petals the visitor probed per visit) and A or S (number of times that the anthers/stigma were/was touched per visit) when large flies visited *Swertia bimaculata* monoclinous flowers. Male‐phase (*N* = 71) and female‐phase (*N* = 62) flower data were used separately to examine P‐A and P‐S correlations. The area of each circle is directly proportional to replicate number

There was a significant positive correlation between P (the number of petals the visitor probed in a visit) and A (the number of times that the anthers were touched in a visit) in large flies on male‐phase flowers (Spearman's ρ = 0.614, *N* = 71, *p* < .01; *r* = .675, regression coefficient = 0.673 ± 0.089 (*SE*); Figure 9). Correlation between P and S (the number of times that the stigma was touched in a visit) in large flies in female‐phase flowers was also significant, with a lower correlation coefficient (Spearman's ρ = 0.471, *N* = 62, *p* < .01) and a lower regression coefficient (0.242 ± 0.050 (*SE*); Figure 9).

**Figure 10:**
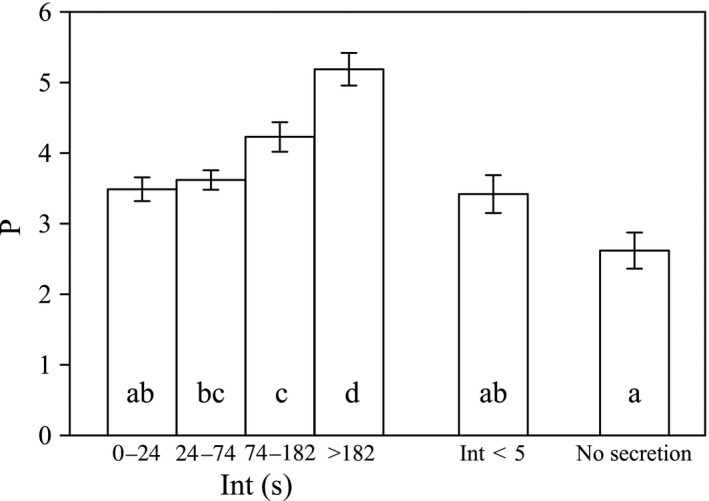
Correlation between P (number of petals the visitor probed per visit) and Int (visiting interval) when insects visited *Swertia bimaculata* monoclinous flowers. Mean values and standard errors (*SE*) of P are displayed using columns and bars, respectively. Visiting interval is defined as the time between the departure of the previous visitor and the arrival of the subsequent visitor. If the previous visitor was still in the flower when the subsequent visitor arrived, Int was regarded as zero. Data from 1,083 visits to monoclinous flowers were collected and used in the first four sets; these data were divided by the three Int quartiles of 24 s, 74 s, and 182 s, so that the four data sets had similar sampling sizes (268, 272, 272, and 271). The fifth data set was drawn from the first set by adjusting the upper limit of the visiting interval to 5 s. The last set of data was collected from 26 visits to post‐female‐phase flowers, whose nectar secretion had completely stopped, disregarding Int. Different annotation letters indicate a statistically significant difference at α = 0.05 (Mann–Whitney test)

There was a significant positive correlation between P and Int (the interval between two visits) in monoclinous flowers, for all visitor species (Spearman's ρ = 0.227, *N* = 1083, *p* < .01) and for large flies (Spearman's ρ = 0.278, *N* = 115, *p* < .01). Thus, as Int increased, a visitor would, on average, visit more petals in a flower (Figure 10).

**Figure 11:**
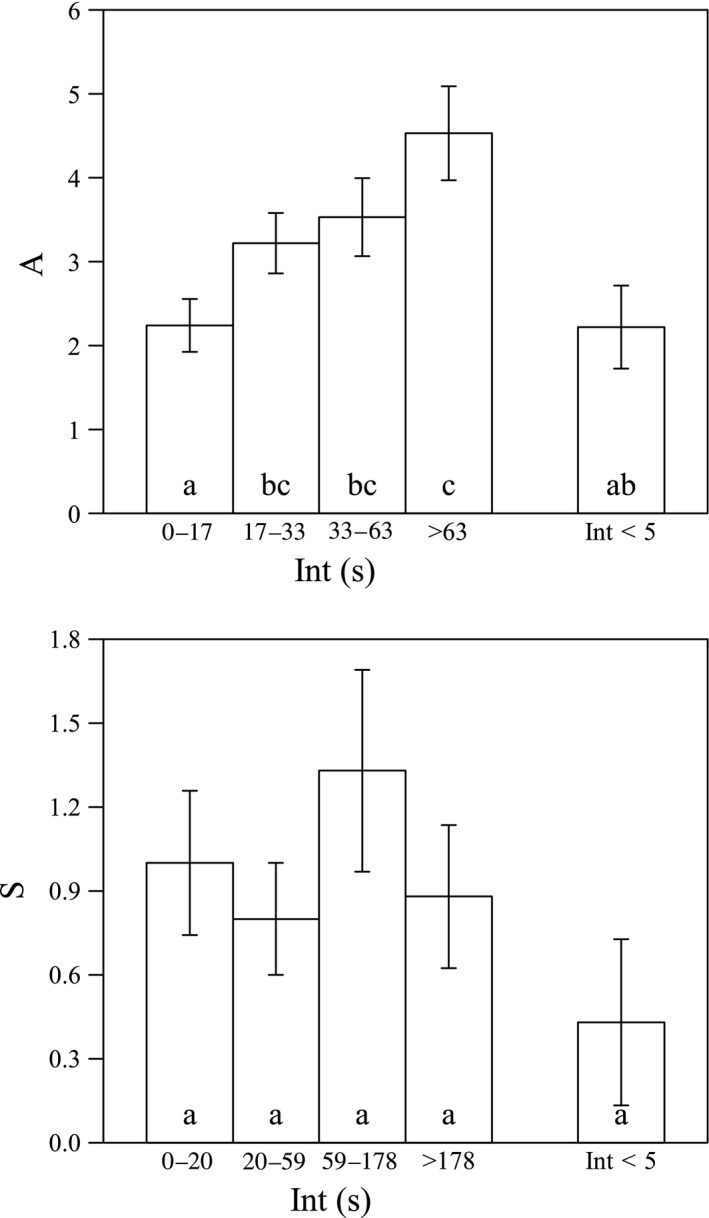
Correlation between A or S (number of times that the anthers/stigma were/was touched per visit) and Int (visiting interval) when large flies visited *Swertia bimaculata* monoclinous flowers. Mean values and standard errors (*SE*) of A or S are displayed using columns and bars, respectively. Visiting interval is defined as the time between the departure of the previous visitor and the arrival of the subsequent visitor. If the previous visitor was still in the flower when the subsequent visitor arrived, Int was regarded as zero. Data from 71 visits to male‐phase flowers and 62 visits to female‐phase flowers were collected and used in the two panels, respectively. Data were divided into four sets by the three Int quartiles (male‐phase flowers: 17 s, 33 s, and 64 s; female‐phase flowers: 19.5 s, 58.5 s, and 178.25 s), so that the four data sets had similar sampling sizes (male‐phase flowers: 17, 18, 17, and 19; female‐phase flowers: 16, 15, 15, and 16). In each panel, the fifth data set was drawn from the first set by adjusting the upper limit of the visiting interval to 5 s. Different annotation letters indicate a statistically significant difference at α = 0.05 (two‐sample *t* test for A and Mann–Whitney test for S)

The correlation between Int and A was significant in male‐phase flowers, for large flies (Spearman's ρ = 0.371, *N* = 71, *p* < .01) and for all visitor species (Spearman's ρ = 0.126, *N* = 636, *p* < .01). No significant correlation between Int and S was found in female‐phase flowers, for large flies (Spearman's ρ = 0.101, *N* = 62, *p* = .435) or for all visitor species (Spearman's ρ = 0.072, *N* = 426, *p* = .136). In other words, in male‐phase flowers, the average pollination effectiveness of a visitor (or a large fly) would increase as Int increased, but in female‐phase flowers, the increment of Int did not lead to a significant increase in an individual insect's pollination effectiveness (Figure 11).

## 4 DISCUSSION

### 4.1 The pollination effects of gland patches: pollinator manipulation and filtration

Two hypotheses were made concerning the possible pollination effects of gland patches: visual attraction and visitor manipulation. Results of the present study rejected the visual attraction hypothesis, because artificially removing or blocking the gland patches did not cause a significant reduction in insect visiting frequency, suggesting that gland patches are not essential to visitor attraction. The visitor manipulation hypothesis, on the contrary, was widely supported by the results. The evidence for this hypothesis was the visitors’ circling movement along the nectary track, which was the most commonly observed behavior mode in *S. bimaculata* visitors. In addition, nectary removal or blockage caused a significant reduction in visit duration, interrupting the circling behavior (field observation showed that most visitors left the non‐nectary flowers after probing approximately two petals, instead of circling on the corolla and passing through approximately 4–5 petals). This indicated that gland patches are essential for encouraging the circling behavior. However, it is important to examine the extent to which the guidance of a visitor's circling behavior relies on available nectar volume (i.e., the nectar standing crop) in the flower and to what extent it relies on a long‐term (“taming”) effect exerted by the flowers formerly visited by the insect (i.e., if some formerly visited flowers had abundant nectar, the insect may tend to probe more petals in a flower with little nectar in the first probed petal than to fly to another flower right after probing the first petal). To answer this question, the correlation between P (the number of petals probed by the visitor in a visit) and Int (the interval between two visits) was examined. Two assumptions were made: each visitor left little nectar behind after visiting a flower, and during Int, the nectar was gradually secreted, so that available nectar volume was positively correlated with Int. The results revealed a significant positive correlation between P and Int in monoclinous flowers, for all visitor species and for large flies. This reflected a positive effect of short‐term food rewards on circling behavior. Nevertheless, the mean value of P remained above three when Int was less than 5 s, and even in post‐female‐phase flowers (the corolla had not closed but nectar secretion had completely stopped, with absolutely no available nectar), the average P was still above two (Figure 10). This showed that there was also a long‐term “taming” effect contributing to the maintenance of circling behavior, especially under a high visiting frequency.

According to the above, nectary tracks play a key role in manipulating the pollinators’ behavior, encouraging them to pass through most of the petals in a flower (instead of a minority of them), reflected in the typical circling behavior. However, it might be more interesting to verify whether promoting circling behavior could benefit the pollination of *S. bimaculata* (i.e., will average pollination effectiveness increase if the pollinator probes more petals in a flower?). Video and field observation showed that when an effective pollinator (e.g., a large fly) fed on nectar on successive petals, one side of the insect's body contacted the anthers successively; thus, numerous pollen grains adhered to the hairy parts of the insect (Figure 1f,g). The significant positive correlation between P and A or S (the number of times that the anthers/stigma were/was touched in a visit) in large flies revealed that promoting P would benefit both pollen dispersal and pollen receipt in *S. bimaculata* (Figure 9), although pollen receipt may be indirectly improved by the circling behavior (i.e., the typical circling behavior itself may not lead to stigma contact, but with more circling movement during a visit, the insect would have a greater chance to exhibit an atypical behavior that would result in stigma contact).

The correlation analysis between Int and A or S was a more direct examination of available nectar volume's effect on pollination effectiveness. The results showed a significant correlation between Int and A in male‐phase flowers, for all visitor species and for large flies (Figure 11), but in female‐phase flowers, no significant correlation between Int and S was found. This suggested that the amount of available nectar mainly facilitated pollen dispersal instead of pollen receipt.

Although the nectaries in *S. bimaculata* monoclinous flowers are closer to the androecium than to the stigma, the nectary–anther distance is not short enough to allow all nectar‐feeding insects to touch the anthers. The results showed that more than 63% of insect visits to male‐phase flowers were ineffective (i.e., no anthers were touched), and there was a positive correlation between pollinator species’ body size and the species’ average pollination effectiveness in male‐phase flowers (Figure 8). Therefore, the combination of nectary track and floral design (e.g., anther location) in male‐phase flowers can be regarded as a spatial filtration mechanism that gives larger visitors a greater chance to contact anthers, reducing pollen waste on smaller visitors who have less chance to contact stigmas in female‐phase flowers (Figure 8). Moreover, owing to stamen movement, the anther–nectary distance gradually shortens during the male phase (Figure 2), which might help to establish a temporal order in pollinator filtration. That is, in the early period of the male phase, the pollen would be transferred almost exclusively by the largest pollinators (who also had the greatest chance to contact stigmas in female‐phase flowers, see Figure 8), and in the late period of the male phase, as the anther–nectary distance shortened, any remaining pollen would be transferrable by pollinators with smaller body sizes. This can be regarded as a strategy to ensure pollen dispersal when the flower was not visited by the largest pollinators in the early period of the male phase.

### 4.2 Gland patch comparison with other types of corolla nectaries

Floral nectaries can occur in virtually all parts of a flower, and they can be divided into receptacular, hypanthial, perigonal, calyx, corolla, androecial, or gynoecial nectaries based on their location (Schmid, 1988). Corolla nectaries (also called petal nectaries) are primarily distributed in Nymphaeaceae, Magnoliales, Ranunculales, Malvales, and Dipsacales (Bernardello, 2007). Unlike in *Swertia bimaculata*, whose nectary and nectar are presented on the middle of petals, corolla nectaries in most other species are located on the basal part of the (inner whorl of) petals (e.g., *Cabomba* spp. in Nymphaeaceae, *Pseuduvaria* spp. in Annonaceae, and *Magnolia* spp. and *Liriodendron* spp. in Magnoliales; Brown, 1938; Huang, Guo, Pan, & Chen, 1999; Thien, 1974; Schneider & Jeter, 1982; Schneider, Tucker, & Williamson, 2003; Silberbauer‐Gottsberger, Gottsberger, & Webber, 2003; Vogel, 1998), on a saccate or spurred part of the corolla (e.g., *Epimedium* spp. in Berberidaceae, *Aquilegia* spp. in Ranunculales, and *Halenia* spp. in Gentianaceae; Suzuki, 1984; Hodges, 1997; Erbar, Kusma, & Leins, 1999; Chassot, Nemomissa, Yuan, & Küpfer, 2001; Von Hagen & Kadereit, 2003), on the adaxial side of the corolla tube (e.g., Caprifoliaceae and Adoxaceae; Brown, 1938; Davis, 2003; Fahn & Rachmilevitz, 1970; Wagenitz & Laing, 1984; Weberling, 1977), or the whole petal is specialized into a small nectariferous organ (previously known as a honey leaf) occurring beside the androecium, whereas petaloid sepals serve as visual attractants (e.g., *Helleborus* and *Trollius* spp. in Ranunculaceae; Cronquist, 1981; Fahn, 1979; Kosuge, 1994; Schmid, 1988; Smets, 1986). Therefore, nectar of these species is usually stored inside spurs, hidden in corolla tubes, or enfolded by other floral structures such as inner petals. In this context, the gland patch is a peculiar form of corolla nectary because it fully exposes nectar on the visually attractive surface. The microscopically rough surface sculpture of the adaxial surface of the *S. bimaculata* corolla revealed in the present study (Figure 4b,c) would generate a “lotus effect”, described as ultrahydrophobicity that makes water form spherical droplets (Barthlott & Neinhuis, 1997). In *S. bimaculata*, this effect might help to ensure that the nectar droplet is restricted to the gland patch region (Figure 1e) instead of spreading over the corolla, which might cause nectar loss and/or confusion in visitor manipulation. According to the literature, plant species with corolla nectaries would attract several groups of insect pollinators, including bees (Huang, Guo, Pan, & Chen, 1999; Suzuki, 1984), beetles (Thien, 1974), and flies (Schneider & Jeter, 1982; Silberbauer‐Gottsberger, Gottsberger, & Webber, 2003); however, none of these reports addresses pollination in species whose nectar is fully exposed on the petal surface, so the present study is important for a comprehensive understanding of the pollination effect of floral nectaries.

### 4.3 Nectar presentation and pollinator preference

In *S. bimaculata*, the fully exposed nectar presentation suggests that short‐tongued pollinators are preferred visitors (Corbet, 2006; Nicolson, 2007), and nectar on the flat surface of the gland patch may be easy to collect by sponging mouthparts that uptake nectar by capillary adhesion rather than suction (Kingsolver & Daniel, 1995; Krenn, Plant, & Szucsich, 2005). These inferences were supported by our records regarding insect behavior and visiting frequency. Field observation showed that, most of the time, only a small volume of nectar was present in a flower because of the high visiting frequency, making bees less efficient at gathering the nectar through their chewing or siphoning mouthparts (Figure 1h). The feeding efficiency of flies, on the contrary, was not affected by the small amount of nectar (Figure 1f,g,j,k; Cruden, Hermann, & Peterson, 1983; Faegri & van der Pijl, 1979).

According to the literature, bees (especially bumblebees) are the main pollinators of most Gentianaceae (Bynum & Smith, 2001; Duan, Dafni, Hou, He, & Liu, 2010; Duan, He, & Liu, 2005; He & Liu, 2004; Kozuharova, Anchev, & Popov, 2003; Oostermeijer, Van Eijck, & Den Nijs, 1994; Petanidou, Den Njjs, & Oostermeijer, 1995; Petanidou, Ellis‐Adam, Den Njjs, & Oostermeijer, 1998). Bees were also reported as the most common floral visitors and frequent pollinators of several *Swertia* species with fimbriate nectaries, such as *S. przewalskii* and *S. chirayita* H. Karst. (Duan & Liu, 2003, 2007; Khoshoo & Tandon, 1963). The present study revealed a significantly higher visiting proportion of flies in *S. bimaculata* than in *S. kouitchensis*. Considering that the populations of the two species grew in almost the same area, it is most likely that the difference in visitor proportion was caused by a difference in floral attraction between the two species, rather than different compositions of insect communities affected by geographical factors. This suggests that flowers with gland patches have a stronger tendency than flowers with fimbriate nectaries to attract flies. Furthermore, at both colonial and individual levels, flies had significantly higher stability and effectiveness than bees in *S. bimaculata*, and large flies behaved far more stably and effectively than other flies and bees in monoclinous flowers. Therefore, it is most likely that flies, especially large ones, are the primary pollinator group of *S. bimaculata*. The effectiveness of large flies in male‐phase flowers is closely related to their body size (Figure 8), and their more variable and random behaviors made them more likely than bees to contact the stigma in female‐phase flowers. These results, which contrast with those reported in other species of Gentianaceae, may imply that a pollinator shift had occurred in *S. bimaculata* along with the transformation of nectary morphology and floral design.

### 4.4 The economics of nectar feeding and nectar production in *S. bimaculata*

Although pollination is usually regarded as a mutualism, there is always a conflict of interest between the plant and the pollinator. Both participants should be trying to balance their costs against the rewards and hence assessing the net benefits gained (Willmer, 2011). Nectar is not only an essential reward to the pollinator but also a substantial cost to the plant (De la Barrera & Nobel, 2004), which makes it a key factor to understand plant–pollinator interactions. From the economic point of view, *S. bimaculata* is “tricky” for presenting a fully dispersed pattern of nectar within a single flower, which makes the visitor tend to move intraflorally instead of interflorally. For a visitor, flying from a petal in one flower to a petal in another flower will cost far more energy than walking to the adjacent petal in the same flower (cf. Voigt & Winter, 1999), whereas the expected reward gained by the two choices is equal. Therefore, it can be hypothesized that a nectar forager can achieve the best energy budget if it feeds on all five petals within a flower before moving to the next flower. Video analysis showed that an average insect (or a large fly) visitor would walk across 4.1 (or 4.2) petals in a monoclinous flower, which is consistent with the previous hypothesis.

The present study showed that a male‐ or female‐phase flower of *S. bimaculata* produced 13.9 or 8.5 μl of nectar per day on average, while the average insect visiting frequency in the two types of flowers was 21.4 and 13.2 times flower^−1^ hr^−1^, respectively. Thus, the mean reward per visit can be estimated. Assuming that the visitors are active for 5 hr per day (based on field observations), the mean reward would be 0.130 μl or 0.129 μl in a male‐ or female‐phase flower. Assuming a constant nectar secretion over 24 hr, if the nectar that accumulated through the night was all taken by the first visitor in the morning, there would only be a mean reward of 0.0271 or 0.0268 μl in a male‐ or female‐phase flower supplied for other visitors throughout the day. These results implied a potential “market mechanism” under which the visiting insects could expect equal rewards of nectar from two different sexual phases of flowers by adjusting their visiting probability (although the actual rewards might differ owing to differences in sugar concentration). According to Faegri and van der Pijl (1979) and Cruden, Hermann, & Peterson (1983), such a level of mean reward is more similar to that of a typical fly‐pollinated generalist flower (less than 0.05 μl) than a bee‐pollinated flower (0.10–10.00 μl). Notably, mean nectar reward volume under natural circumstances may differ from our estimates, which were based on daily measurements from bagged flowers. Under natural circumstances, nectar would be removed far more frequently (than daily removal) by floral visitors. The literature suggests that removing nectar more frequently may accelerate or decelerate nectar secretion, or it may have no effect (Bernardello, Galetto, & Rodriguez, 1994; Corbet, 2003; Cruden, Hermann, & Peterson, 1983; Galetto & Bernardello, 1992, 1993, 1995; Galetto, Bernardello, Isele, Vesprini, Speroni, & Berduc, 2000; Galetto, Bernardello, & Juliani, 1994; Ornelas, Ordano, & Lara, 2007; Pyke, 1991; Rivera, Galetto, & Bernardello, 1996; Torres & Galetto, 1998; Vesprini & Galetto, 2000).

The common range of nectar sugar concentration (C) measured in temperate flowers is 20%–50% (Willmer, 2011), and for flowers with fully exposed nectar, the C can be even higher due to the rapid evaporation of the water content in dry air (Corbet, Unwin, & Prys‐Jones, 1979; Corbet, Willmer, Beament, Unwin, & Prys‐Jones, 1979; Willmer, 1983). The present study, however, revealed C that averaged 7.6%. Producing nectar with such a low C may be a strategy to prevent the nectar from becoming too concentrated and viscous for the pollinators to ingest (cf. Corbet, Unwin, & Prys‐Jones, 1979; Willmer, 1983). It is also possible that the C was diluted to some extent by moist air or dew in the mornings during the investigation.

According to De la Barrera and Nobel (2004), the production of nectar often peaks with maximum pollen availability, but sometimes it peaks with the maximum stigma receptivity; male fitness is often more strongly associated with nectar production than is female fitness (Aizen & Basilio, 1998; Mitchell, 1993; Pleasants & Chaplin, 1983). Carlson (2007) found that the nectar accumulation rate (indicated by sugar mass) of *Chrysothemis friedrichsthaliana* (Hanst.) H. E. Moore (Gesneriaceae) was greater during the male phase than the female phase, with a difference of 83%. The present study showed a similar result in that the volume, concentration, and sugar content of nectar produced daily by a *S. bimaculata* flower were all higher in the male phase than in the female phase, with a difference of 64%, 74%, and 139%, respectively. Moreover, both daily nectar concentration and daily nectar sugar content declined significantly at the beginning of the female phase, indicating a male‐biased nectar production schedule.

The present study revealed that, in *S. bimaculata*, nectar rewards were usually very abundant in only a few flowers (see the outliers in Figure 5). This might represent an evolutionarily stable strategy to produce both nectarful and nectarless flowers within a plant species; wherein, the cost to the plant can be minimized through automimic flowers that produce no nectar (Bell, 1986; Brink, 1982; Thakar, Kunte, Chauhan, Watve, & Watve, 2003; and references in Gilbert, Haines, & Dickson, 1991).

### 4.5 Nectar production and floral design tend to enhance male function in *S. bimaculata*

A widely tested theory in pollination ecology is that in plants with monoclinous flowers, the evolution of floral attractive traits may be driven primarily by selection on male function because increased pollinator visits may be more beneficial to male function than female function (Aizen & Basilio, 1998; Bell, 1985; Burd & Callahan, 2000; Campbell, 1989; Carlson, 2007; Devlin & Stephenson, 1985; Galen & Stanton, 1989; Lloyd & Yates, 1982; Melendez‐Ackerman & Campbell, 1998; Stanton, Snow, & Handel, 1986; Willson, 1994), based on the assumption that male fitness is most strongly limited by access to mates, whereas the strongest limiting factor on female fitness is usually resources (Bateman, 1948; Charnov, 1979; Darwin, 1871). The present study indicated that *S. bimaculata* supports this theory: The manipulatory effect of the nectary track nearly exclusively promoted the chance of anther contact, not stigma contact, showing a male‐biased floral design. The nectar production schedule is also male‐biased, implying a male‐biased resource allocation regarding floral rewards. Furthermore, the male‐phase flowers had a higher frequency of insect visits and effective visits and a greater probability of effective visits than female‐phase flowers, which are correlated with the male‐biased floral design and nectar production schedule.

In *S. bimaculata*, the bias toward male function may have made the gynomonoecious sexual system an evolutionarily stable strategy (cf. Charnov, Smith, & Bull, 1976). In female flowers, the abortion of stamens could eliminate interference from the androecium, hence facilitating the female flowers achieving greater female fitness than monoclinous flowers with an equal investment of ovule resources (i.e., higher seed‐set rates). The results of this study provide evidence for this inference, as the natural seed‐set rate in female flowers was significantly higher than that in monoclinous flowers, whereas the female flowers had significantly lower visiting frequencies.

### 4.6 Comparison of pollination in *S. bimaculata* and plants with generalist/specialist pollination

Both the open design of the corolla and the fully exposed nectar presentation in *S. bimaculata* suggested a generalist pollination syndrome (Corbet, 2006; Faegri & van der Pijl, 1979). Pollination in generalist flowers has been described as “catering for the mass market” by Proctor, Yeo, and Lack (1996) or “mess pollination” by Willmer (2011). This concept was supported by the numerous species of floral visitors observed in the present study. Nevertheless, almost all visiting insects exclusively exhibited circling behavior on *S. bimaculata* flowers, reflecting an essential difference from mess pollination, in which case the visitors scrabble on the flower or inflorescence in a disorderly manner. In classically generalist flowers, pollen is easily accessible for most floral visitors, whereas, in *S. bimaculata*, most insect visitors were too small to contact the anthers or stigma while feeding on nectar. Based on the comparison made above, it could be derived that *S. bimaculata* flowers were pollinated in a more ordered and selective way than typical generalist flowers. Furthermore, nectary tracks, which were not observed in generalist flowers, were essential in manipulating and filtering the visitors. Thus, pollination in *S. bimaculata* does not fit a generalist syndrome.

Notably, the selective strategy used by *S. bimaculata* in pollinator filtration was quite different from that used by specialist flowers. Specialist flowers usually present a specific syndrome, ensuring that only the specific pollinators can be attracted to the flowers and access pollen and/or nectar (Baker & Hurd, 1968; Faegri & van der Pijl, 1979; van der Pijl, 1961), whereas, in *S. bimaculata*, various insect species are attracted to the flowers owing to the easily accessible food reward, although the majority of them have little chance to contact the anthers. In other words, the filtration process for effective pollinators is “prearrival” in specialist species and “postarrival” in *S. bimaculata*.

### 4.7 The mating strategy of *S. bimaculata*, in comparison with other reported Gentianaceae spp.

It is widely known that organisms with sexual reproduction benefit from outcrossing: Outcrossed progeny is expected to be more heterozygous, hence individually more adaptive and with more fitness than selfed progeny (Agrawal, 2006; Bell, 1982; Maynard Smith, 1978). However, in flowering plants, pure outcrossing (e.g., self‐incompatible) may bring potential risks to the number of offspring because cross‐fertilization relies on an external agent for pollen transfer, for example, animals and wind, which are often unpredictable and/or inadequate (Eckert, Samis, & Dart, 2006). This is likely why the majority of modern plant species evolved toward a mixed mating strategy, that is, a mixture of selfing and outcrossing (Goodwillie, Kalisz, & Eckert, 2005; Harder & Barrett, 1996; Vogler & Kalisz, 2001). In Gentianaceae, the plants are generally self‐compatible (Duan & Liu, 2003; Duan, Dafni, Hou, He, & Liu, 2010; Dudash, 1993; Fischer & Matthies, 1997; Freitas & Sazima, 2009; He & Liu, 2004; Lennartsson, Oostermeijer, van Dijk, & den Nijs, 2000; Oostermeijer, Van Eijck, & Den Nijs, 1994; Petanidou, den Njjs, & Oostermeijer, 1995; Petanidou, Ellis‐Adam, den Njjs, & Oostermeijer, 1998, 2001), and protandry (Bynum & Smith, 2001; Duan & Liu, 2003; Duan, He & Liu, 2005; Dudash, 1993; Oostermeijer, Van Eijck, & Den Nijs, 1994; Petanidou, Den Njjs, & Oostermeijer, 1995; Petanidou, Ellis‐Adam, Den Njjs, & Oostermeijer, 2011; Webb & Littleton, 1987; Webb & Pearson, 1993) and herkogamy (Bynum & Smith, 2001; Duan & Liu, 2003; Duan, Dafni, Hou, He, & Liu, 2010; Duan, He, & Liu, 2005; Lennartsson, Oostermeijer, van Dijk, & den Nijs, 2000; Webb & Pearson, 1993) are frequently reported mechanisms for avoiding autogamy and sexual interference. Nevertheless, highly selfed mating system has evolved along with the loss of protandry and/or herkogamy in some Gentianaceae spp. (Fischer & Matthies, 1997; Machado, Sazima, & Sazima, 1998; Webb & Pearson, 1993), probably in response to the pollen limitation caused by unstable pollinator abundance and/or constancy (Duan, Zhang, & Liu, 2007; Dudash, 1993; Petanidou, den Njjs, & Oostermeijer, 1995; Petanidou, Ellis‐Adam, den Njjs, & Oostermeijer, 1998). Some Gentianaceae spp. grown in harsh habitats have developed mechanisms of spontaneous self‐pollination as a delayed mechanism for reproductive assurance (Duan, Dafni, Hou, He, & Liu, 2010; Freitas & Sazima, 2009) or even as a main contributor to natural seed set (Petanidou, Ellis‐Adam, den Njjs, & Oostermeijer, 1998). In the present study, floral lifespan and morphology investigations showed that *S. bimaculata* was both protandrous and herkogamous (herkogamy was especially distinct in the female phase), and the pollen dispersal experiment confirmed the strictness of dichogamy, as the free‐pollinated anthers were normally emptied by pollinators in less than one day, long before the exposure of stigma. According to these results, it is most likely that autogamy (selfing within a flower) does not occur in the *S. bimaculata* population under natural circumstances. Thus, this species can only be pollinated by insect pollinators with geitonogamous or xenogamous pollen. The occasional fruit set of the bagged flowers in the mating system experiment was probably caused by accidental self‐pollination, where the bag was blown by the wind, causing successive contact with anthers and stigma. During the field experiment, in the female phase of these bagged flowers, most pollen was still adhered to the anthers or the inner surface of the bag because insect visitors were excluded. The P/O ratio coefficient also suggested a highly outcrossed mating system, probably with a relatively high outcrossing rate among Gentianaceae species; according to Duan and Liu (2007), the P/O ratio of *S. przewalskii* is approximately 250–300, which is much lower than that of *S. bimaculata* (approximately 900–1000). Despite the strict prevention of self‐pollination, there were considerable fruit‐ (over 90%) and seed‐set rates (over 80%) in the free‐pollinated *S. bimaculata* flowers. Furthermore, no significant pollen limitation was revealed, suggesting that being both protandrous and herkogamous was a successful mating strategy for *S. bimaculata* because it helped the species to produce offspring with both quality (highly outcrossed) and quantity (high seed‐set rate). The possibility of being highly outcrossed without losing seed set is based on the premise of stable, effective, and ordered pollination, owing to high pollinator abundance in the habitat and the extraordinary design of nectary tracks.

## CONFLICT OF INTEREST

None declared.

## AUTHOR CONTRIBUTIONS

Shuai Wang addressed main questions, designed major experiments, participated in data acquisition, analyzed and interpreted data, and drafted the article. Wen‐Long Fu contributed to conception and experiment design, participated in data acquisition, and critically revised the article for important intellectual content. Wei Du drew initial attention to the target species, contributed to conception, and critically revised the article for important intellectual content. Qi Zhang participated in data acquisition and critically revised the article for important intellectual content. Ya Li participated in data acquisition and critically revised the article for important intellectual content. Yu‐Shu Lyu participated in data acquisition and critically revised the article for important intellectual content. Xiao‐Fan Wang (the corresponding author) addressed main questions, contributed to conception, and critically revised the article for important intellectual content.
